# Climate change on YouTube: A potential platform for youth learning

**DOI:** 10.34172/hpp.2020.42

**Published:** 2020-07-12

**Authors:** Beatriz Duran-Becerra, Grace C. Hillyer, Alison Cosgrove, Corey H. Basch

**Affiliations:** ^1^Herbert Irving Comprehensive Cancer Center, Columbia University, NY, NY 10032, USA; ^2^Department of Epidemiology, Mailman School of Public Health, Columbia University NY, NY 10032, USA; ^3^Department of Public Health, William Paterson University, Wayne, NJ 07470, USA

**Keywords:** Climate change, Social media, Adolescents

## Abstract

**Background:** Climate change is one of the most critical threats to our society. The purpose of this cross-sectional study was to describe the content of the most viewed climate change videos on YouTube.

**Methods:** The term "climate change" was used to search on YouTube to garner a sample of the 100most widely-viewed videos. Videos in a language other than English, or considered irrelevant, were excluded. Using a fact sheet from National Aeronautics and Space Administration, content categories were created and successively coded.

**Results:** The mean number of views for the 100 videos evaluated was 231,140.2 views (SD=718, 399.5) and the mean length was 12.1 minutes (SD= 24.1). Most videos were uploaded by a news source (77.0%), included a belief that climate change is happening (77.0%), and mentioned the impact of climate change on the environment (71.0%). Only one-third of the videos mentioned how to prevent climate change (33.0%). More than half focused on a specific environment and, of those, 47.2% specifically focused on cities. Compared to videos that did not focus on a specific environment, the videos with an environmental focus were more often intended for adults (87.3% vs. 53.3%, P≤0.001).

**Conclusion:** This study highlights the need for climate change YouTube videos intended for youth. Targeting youth may lead to engagement of younger generations in climate change discourse and inspire climate action. Further research is needed to determine the effectiveness of YouTube as a platform for educational videos on climate change.

## Introduction

Climate change is globally established as one of the most critical threats to our society. According to the Fourth National Climate Assessment report, the global climate will continue to change over the next century and the magnitude of change will depend primarily on emission of greenhouse gases emitted by human activity.^1^ The impacts of climate change include an uptick in extreme weather events, disruption of water access and food security, damage to biodiversity, rising temperatures, and harm to human health.^1-4^ Even though climate change is a universal threat, minority and low-income communities disproportionally face the environmental, economic, and health consequences.^5-8^ Increasing public engagement is essential in mitigating climate change through proper climate change education, especially among the younger generations who will primarily experience the consequences of current human activity.

Although a majority of Americans believe that protecting the environment and dealing with climate change should be a priority, beliefs about causal factors remain polarized among the general public.^9^ This polarization is significant due to the large-scale changes in individual behaviors and policy needed to address climate change. Many researchers have proposed that lack of action and engagement may also be due to people’s perception of climate change as distant in time and space.^10^ Research suggests that communicating the observable effects of climate on specific places could increase public engagement and action in climate change efforts.^11,12^

Social media websites have gained global popularity as important information sources for topics such as science and medicine.^13^ Ranked as one of the most visited websites, YouTube provides easily accessible information for over 2 billion users of varying science literacy levels and ages.^14^ While there are some studies looking at the association between social media and public awareness and engagement with climate change, there is not extensive research on the type of information available on YouTube regarding climate change.^15,16^ The purpose of this study was to describe the content of the most viewed climate change videos on YouTube.

## Materials and Methods

This study used a cross-sectional design to evaluate the content of climate change videos on YouTube. The term “climate change” was used to search on YouTube to garner a sample of the 100 most widely-viewed videos. This was done by filtering for view count and assessing for relevancy. All videos that were not in the English language and were deemed irrelevant because they did not pertain to global climate change were excluded. Using a fact sheet from National Aeronautics and Space Administration,^17^ content categories were created along with determining the purpose or type of the of video, whether or not reliable, scientific sources were cited in video and, the intended audience, characteristics of the person delivering the message. Remaining categories included whether or not the video contained the following: a belief climate change is occurring, discussion of climate change prevention, explanation of climate change trends, the extent to which history of climate change is discussed, explanation of dangers to humans, impact on environment, and a focus on a certain biome.

The options for source of video included American news clip, international news clip, credible scientific source, student project, or other. News clips involve clips from news channels that are derived either from a US or international station. A credible scientific source would be a recognized organization in the scientific community, whereby a student project could be something a student made, or a recording of a project at a college campus. All videos in the other category included videos not fitting into existing categories such as animations or an on-line advanced placement (AP) class available to the public. Sources cited in video indicates if information, quotes, or graphics are cited either in a caption or verbally by the presenter to back up claims. This was determined by evaluating sources, if they were present.

The intended audience was assessed to be adults, children, students, or not applicable. This was determined by analyzing the type of video, the language used, and how information was presented. A video was considered to be for an adult audience if the content was political. In these clips, adults were typically discussing the issue of climate change amongst each other. An example would be a video featuring a man alone discussing the UN report on climate change. A video for children featured more colorful images, simple animations, or if children were present in the video. For example, a short children’s video that featured children answering how they would solve climate change. Videos for students were videos with content educating viewers. For example, a video posted by a teacher to exclusively teach his AP Environmental Science class, or one which explains political ideas, however it is in simple terms and has many images which anyone can follow.

The presence of climate change prevention methods was to ascertain if the video was only stating the problem of climate change, or if it was actively informing viewers on how to combat the changes. In order to determine the extent to which a video focuses on the history and trends of climate change, the following steps were taken. If more than 75% of the video explained changes which have occurred or are occurring, it was considered a high amount. If 50%-74% of the video discussed changes, it was a moderate amount. A low amount would be considered 25% to 49%. In the cases where trends were not discussed at all, videos this category was marked not applicable. Dangers to human beings and dangers to the environment were important categories to determine focal points of the video.

Videos which focused on dangers to humans, explaining the perils climate change creates for specific populations, which vary by video. In some cases, health was not discussed in terms of disease. For example, a video that explores what the outcome of the community would be if many people became homeless due to the rising water level. Additionally, this category included videos which described impacts on the environment, as well as solutions. For example, a video of a politician acknowledging what is causing harm to the environment in Canada then end by explaining a beneficial intervention.

In order to describe if the video focused on a certain place or environment, each video was coded for mention of the following categories were rainforests, arctic, cities/suburbs, deserts, plains, other, not mentioned, and oceans. The “other” option was used when the planet was explained very broadly, or when climate marches were the main emphasis of the video.

The final category reports how information was conveyed or delivered in the video. The options were news reporter, researcher, expert in field, animations, politicians, celebrity, or other. For this study, a researcher was considered to be one who is working on a specific related project that they are speaking on, whereas an expert in field is someone who is recognized for broad, credible research, or someone who has extensive education and experience on the topic. It should be noted that Greta Thunberg, a young climate activist, was considered a celebrity advocate for the purpose of this study as she does not have any formal education on the topic.

### Quantitative content analysis

Categorical data were expressed as frequencies and percentages whereas continuous data were expressed as mean, median, standard deviation, and range. In order to compare videos with a focus on a specific environment and videos without a focus on a specific environment, univariable analysis was conducted using a chi-square test for categorical data and an independent t-test for continuous data. The inter-rater reliability was determined (Cohen’s kappa = 0.929). *P* values <0.05 were considered statistically significant. Quantitative content analysis was conducted using IBM SPSS Statistics software, version 26.^18^ This study did not include any human research subjects and qualified for IRB review exemption.

## Results

The mean number of views for the 100 videos evaluated was 231,140.15 views (SD = 718, 399.5) and the mean video length was 12.1 minutes (SD = 24.1) (Table 1). A majority of the videos were uploaded by a news source (77.0%). The content of most videos included a belief that climate change is happening (77.0%) and mentioned the impact of climate change on the environment (71.0%). However, only a few videos mentioned how to prevent climate change (33.0%). More than half of the videos focused on a specific environment and, of those, 47.2% specifically focused on cities. Compared to videos that did not focus on a specific environment, the videos with an environmental focus were more often intended for adults (87.3% vs. 53.3%, *P* ≤ 0.001). Environment-focused videos also more often had a “high” emphasis on the history and trends of climate change (58.2% vs. 26.7%, *P* = 0.001), explained the impact of climate change on the environment (83.6% vs. 55.6%, *P* = 0.002), and mentioned the dangers of climate change to humans (54.5% vs. 31.1%, *P* = 0.02). Videos without a specific environmental focus more often were geared toward a non-specific audience (35.6% vs. 7.3%, *P* ≤ 0.001) and more frequently were presented by a politician or celebrity (28.9% vs. 5.5%, *P* = 0.001) than those that concentrated on a particular ecological environment.

## Discussion

Content analysis of the 100 most popular YouTube videos on climate change indicated significant differences in content between videos focused on a specific environment and videos that did not focus on a specific environment. Compared to the videos that did not focus of a specific environment, videos focused on a specific environment provided additional information on the history and trends of climate change, the impact of climate change on the environment, and the dangers of climate change to humans. While most videos supported scientific consensus that climate change is happening and mentioned its impact on the environment, most videos did not indicate how to prevent it. Additionally, most videos were uploaded by a news source and were intended for adults. A study assessing if videos on YouTube adhered to scientific consensus on climate change discovered similar results. Allgaier^19^ determined that the majority of popular YouTube videos yielded by the search term “climate change” were uploaded by a TVs news program/documentary and supported scientific views on climate change.

Given the psychological and social barriers to public engagement in climate change action, compelling communication of climate change messages is imperative.^20^ Despite the predicted and current negative impacts of human activity, climate change is not unanimously perceived as a salient issue. Instead, some individuals believe that climate change will only affect future generations and communities outside of their own.^11,21^ Even when people perceive climate change as a pressing issue, they may not feel confident in their ability to change their behavior or may not identify the actions to successfully mitigate the impact of human activity.^22-24^ Framing messages in a way that makes climate change personally relevant and increases individuals’ confidence in addressing it could help with policy support and collective behavior change. Researchers have proposed that localizing the effects of climate change, such as explaining how a specific community or environment is affected, could change the perception of this issue as a distant event.^11,25^ Although research suggests only certain audiences may be perceptive to place-based messages, the frame of climate change messages can influence the type and level of action people are willing to take against climate change.^26-28^ As demonstrated in our study, YouTube videos focused on a specific environment are framed in a way that provides more thorough information on the dangers of climate change. Further research needs to be conducted in order to evaluate the effects of localized climate change messages via social media platforms, such as YouTube, on public engagement.

Furthermore, this study highlights the need for climate change YouTube videos intended for youth. In recent years, the rapidly changing technological landscape and the increasing use of smartphones has expanded access to information and social media platforms among adolescents. YouTube’s growing popularity among ages 13 to 17 underlines the potential of this social media platform to reach and engage youth in critical climate change issues at a large scale.^29^ Growing research emphasizes the value of directly engaging youth in efforts to prevent and adapt to climate change issues.^30,31^ Youth participation in climate change action faces multiple barriers, such as a lack of sense of urgency and a psychological distance from the consequences of climate change.^32,33^ Accurate information on the science of climate change does not overcome these challenges and does not lead to effective engagement.^32^ Studies show youth connection with climate change issues could be accomplished through social media, digital technology, and peer-to-peer communication.^34,35^ Accessibility to platforms such as YouTube encourages youth to create content, share personal narratives, and interact through peer-to peer communication, therefore potentially leading youth to engage in climate change action.^36^

The limitations of this study include the relatively small sample size, cross-sectional design, and the use of English language videos only. In spite of these limitations, this study is one of the first to extensively examine the content of the most popular YouTube climate change videos and to indicate a lack of videos created for youth. A majority of research on climate change perspectives and engagement has focused on adults and school programs, therefore future studies should focus on analyzing the content of climate change videos intended for youth on social media platforms. Analysis of climate change videos targeting adolescents could provide further insight to the type of content needed to successfully engage youth in climate change discourse. This will become particularly important as social media platforms continue to gain more popularity and influence among the younger generations.

## Ethical approval

This study did not include any human research subjects and qualified for IRB review exemption. The Institutional Review Board at William Paterson University does not review studies that do not involve human subjects.

## Competing interests

The authors do not have any potential conflicts of interest to disclose.

## Funding

Funding was not received for this study.

## Authors’ contributions

CHB proposed the research idea and developed the methods for this study. AC and CHB compiled the videos, created content categories, and analyzed videos based on these categories. BDB and GCH analyzed and interpreted the data. BDB developed the initial draft of the manuscript and acted as corresponding author. All authors revised and contributed to the final draft of the manuscript.

**Table 1:** Comparison of video characteristics by focus on a specific environment.

	**Focus on a specific environment**			
	**Total (n=100)**	**Yes (n = 55)**	**No (n = 45)**	***P*** **value**
**Video Characteristics**				
Number of views				
Total	23114015	11149025	11964990	
Mean [SD]	231140.15 [718399.5]	202709.55 [637910.5]	265888.7 [812133.6]	0.44
Median	38,236	39,465	36842	
Range	716-5222618	716-4490919	1174-5222618	
Video length (min)				
Total	1209.5	565.3	644.2	
Mean [SD]	12.1 [24.1]	10.28 [15.8]	14.32 [31.5]	0.10
5.2	
Range	0.90-200.4	0.98-88.8	0.90-200.4	
Video source				
News				0.38
American	33 (33.0)	18 (32.7)	15 (33.3)	
International	44 (44.0)	27 (61.4)	17 (37.8)	
Scientific and other sources	23 (23.0)	10 (18.2)	13 (28.9)	
Intended audience				
Adults	72 (72.0)	48 (87.3)	24 (53.3)	<0.001
Children	28 (28.0)	2 (3.6)	5 (11.1)	0.15
Students	17 (17.0)	9 (16.4)	8 (17.8)	0.85
Non-specific	20 (20.0)	4 (7.3)	16 (35.6)	<0.001
Presentation style				
Presenter				
News reporter	38 (38.0)	27 (49.1)	11(24.4)	0.01
Researcher/expert	23 (23.0)	16 (29.1)	7 (15.6)	0.11
Politician/celebrity	16 (16.0)	3 (5.5)	13 (28.9)	0.001
No presenter, animation	26 (26.0)	10 (18.2)	16 (35.6)	0.05
Sources of information cited				0.22
Yes	33 (33.0)	21 (38.2)	12 (26.7)	
No	67 (67.0)	34 (61.8)	33 (73.3)	
Environment discussed				
Yes	55 (55.0)	55 (100.0)	0 (0.0)	
Artic	8 (8.0)	8 (14.5)	--	
City	26 (26.0)	26 (47.2)	--	
Desert	7 (7.0)	7 (12.7)	--	
Ocean	12 (12.0)	12 (21.8)	--	
Plains	9 (9.0)	9 (16.4)	--	
Rainforest	8 (8.0)	8 (14.5)	--	
Other	12(12.0)	12(21.8)	--	
No	45 (45.0)	0 (0.0)	45 (100.0)	
**Video Content**				
Belief that climate change is happening				0.29
Yes	77 (77.0)	45 (81.8)	32 (71.1)	
No	9 (9.0)	5 (9.1)	4 (8.9)	
Not applicable	14 (14.0)	5 (9.1)	9 (20.0)	
Mentions how to prevent climate change				0.72
Yes	33 (33.0)	19 (34.5)	14 (31.1)	
No	67 (67.0)	36 (65.4)	31 (68.9)	
Focus on explaining climate change history and trends				0.001
High	44 (44.0)	32 (58.2)	12 (26.7)	
Moderate	27 (27.0)	15 (27.3)	12 (26.7)	
Low	21 (21.0)	7 (12.7)	14 (31.1)	
None	8 (8.0)	1 (1.8)	7 (15.6)	
Mentions impact of climate change on the environment				0.002
Yes	71 (71.0)	46 (83.6)	25 (55.6)	
No	29 (29.0)	9 (16.4)	20 (44.4)	
Mentions dangers of climate change to humans				0.02
Yes	44 (44.0)	30 (54.5)	14 (31.1)	
No	56 (56.0)	25 (45.5)	31 (68.9)	
